# Credit to Whom Credit Is Due

**Published:** 1860-09

**Authors:** 


THE department of the REVIEW devoted to "Bi-monthly Abstracts," was instituted with the view of furnishing a comprehensive sketch of the most important contributions to medical periodical literature, both American and European. We have always regarded it as an interesting feature, and have been at considerable expense in placing it in the hands of gentlemen whom we have deemed competent to undertake the task of condensation — a task, we may remark, which, when properly accomplished, is never unattended with trouble and labor. We are glad to see that other journals, both Transatlantic and Cisatlantic, have considered our abstracts worthy of being copied, but regret that they have almost always failed to ascribe to us the credit which we think we honestly deserve. In scarcely more than a single instance do we find any recognition of the NORTH AMERICAN MEDICO-CHIRURGICAL REVIEW, as the source from which the condensed article was extracted. We have lately had presented to our notice half a dozen cases, in which the original journal, from which our own abstract had been carefully prepared, receives the full award, and in one instance, we notice that one of our abstracts is copied into another periodical, which received credit for it, when recopied, as if it were the original authority. We acknowledge a certain amount of gratification that this feature of our journal so frequently meets with attention, but fairly deserve, we think, some recognition of our own efforts to make this department a feature, and of the mode in which the work has been accomplished by the gentlemen to whom the labor of condensation has been intrusted.

